# Supplementary material for: Cell cycle arrest induces lipid droplet formation and confers ferroptosis resistance
Source: Nat Commun. 2024 Jan 2;15:79. doi: 10.1038/s41467-023-44412-7 (PMC10761718; doi:10.1038/s41467-023-44412-7)
Supplement: Supplementary file 1 — Supplementary Information [file 41467_2023_44412_MOESM1_ESM.pdf]

Supplementary Information

Cell-cycle arrest induces lipid droplet formation and confers ferroptosis resistance

Table of contents

Supplementary Figure 1-6
Supplementary Table 1

Supplementary Figures

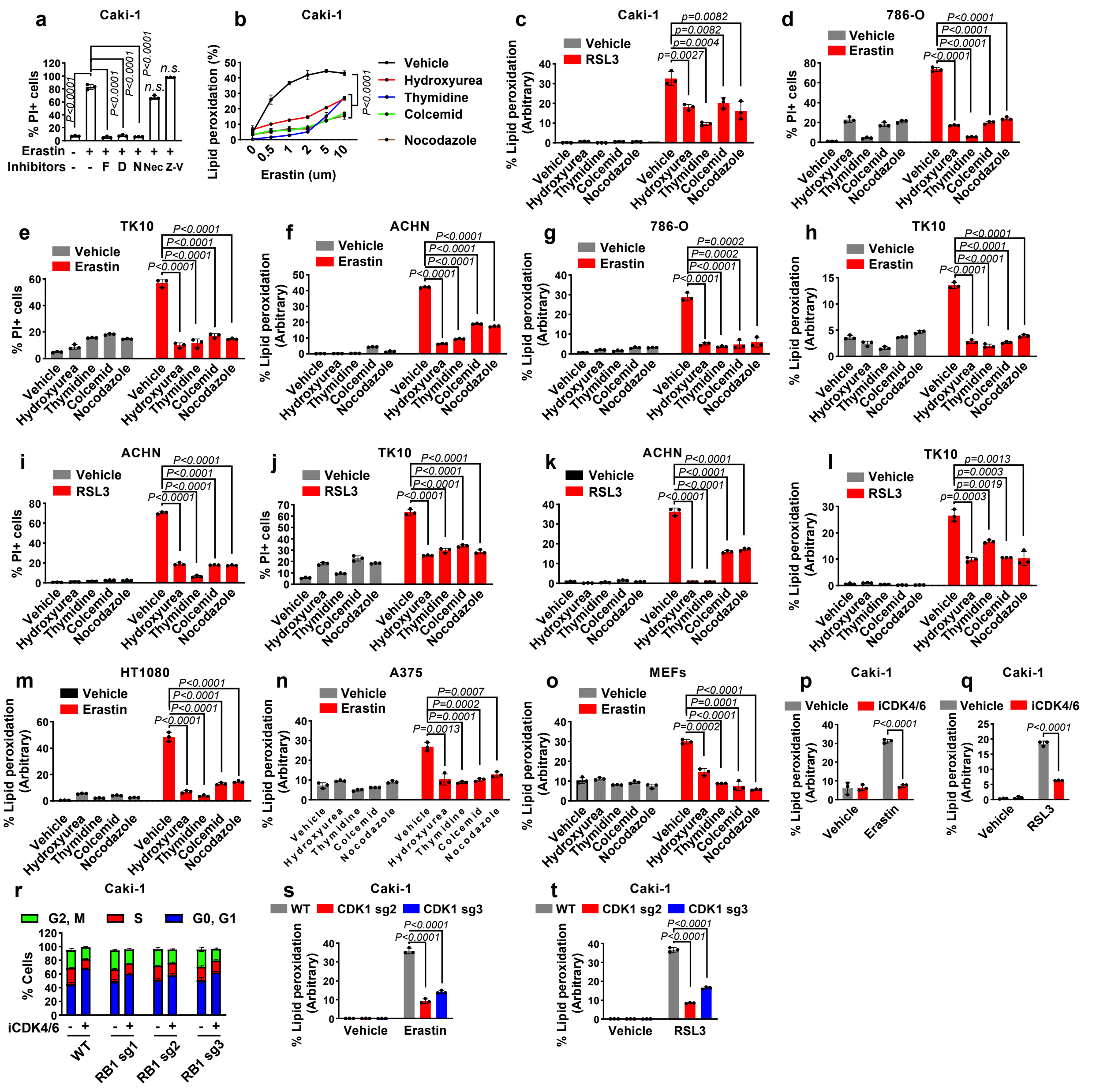

Supplementary Fig. 1. Cell-cycle arrest drives resistance to ferroptosis. a, Populations of PI-positive Caki-1 cells after treatment with 2 μM erastin and cell death inhibitors for 24 h. *n.s.*, not significant; F, 1 μM ferrostatin-1; D, 100 μM DFO; N, 5 mM *N*-acetylcysteine; Nec, 2 μM necrostatin-1s; Z-V, 20 μM Z-VAD-FMK. **b and c**, Lipid peroxidation measurement in Caki-1

cells after 24 h of pretreatment with cell-cycle inhibitors followed by treatment with 0-10 μM erastin for 8 h (**b**) or 25 nM RSL3 for 16 h (**c**). **d and e**, PI-positive 786-O (**d**) and TK10 (**e**) cell populations after 24 h pretreatment with cell-cycle inhibitors followed by 10 μM erastin for 24 h. **f-h**, Lipid peroxidation measurement in cells after 24 h pretreated with cell-cycle inhibitors followed by erastin for 18 h. Erastin, 2 μM in ACHN (**f**), 5 μM in 786-O (**g**), and 5 μM in TK10 (**h**). **i and j**, PI-positive populations of ACHN cells treated with 500 nM RSL3 for 24 h (**i**) and TK10 cells treated with 75 nM RSL3 for 24 h (**j**). **k-o**, Lipid peroxidation measurement in ACHN cells treated with 100 nM RSL3 for 18 h (**k**), TK10 cells treated with 30 nM RSL3 for 18 h (**l**), HT1080 cells treated with 5 μM erastin for 18 h (**m**), A375 cells treated with 10 μM erastin for 18 h (**n**), and MEFs treated with 2 μM erastin for 8 h (**o**). **p and q**, Lipid peroxidation measurement in Caki-1 cells treated with iCDK4/6 and 2 μM erastin for 8 h (**p**) or 25 nM RSL3 for 16 h (**q**). **r,** Cell-cycle profiles for WT and RB1 sgRNA-infected Caki-1 cells. **s and t**, Lipid peroxidation measurement in WT, CDK1 sg2, and CDK1 sg3 Caki-1 cells treated with 2 μM erastin for 8 h (**s**) or 25 nM RSL3 for 16 h (**t**). Mean (± SD) values are shown. $n = 3$. n indicates independent repeats, P values were calculated using two-way ANOVA (**b**) or an unpaired, two-tailed t-test. Source data are provided as a Source Data file.

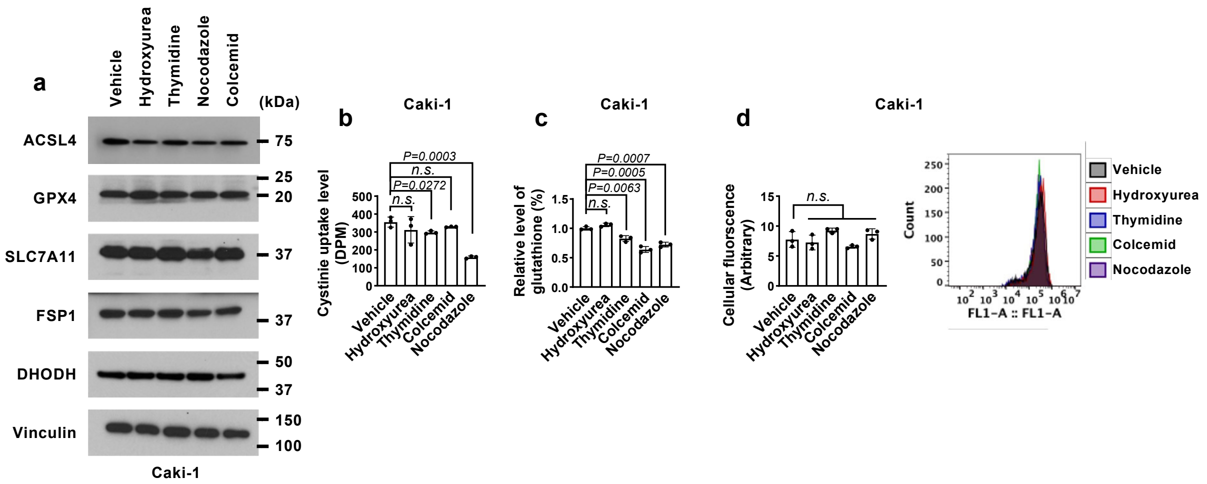

Supplementary Fig. 2. Cell-cycle arrest does not alter biochemical or genetic hallmarks of ferroptosis. a, Immunoblot of the expression of ACSL4, GPX4, SLC7A11, FSP1, and DHODH in Caki-1 cells treated with a vehicle, hydroxyurea, thymidine, nocodazole, or colcemid for 48 h. **b,** Measurement of cystine uptake in Caki-1 cells treated with the indicated vehicle or cell-cycle inhibitors. **c,** Glutathione measurement in Caki-1 cells treated with a vehicle or cell-cycle inhibitors. **d,** Bar graph and histogram showing the levels of intracellular labile iron in Caki-1 cells. Mean (± SD) values are shown. *n* = 3. *n* indicates independent repeats (an unpaired, two-tailed *t*-test). n. s. not significant. Source data are provided as a Source Data file.

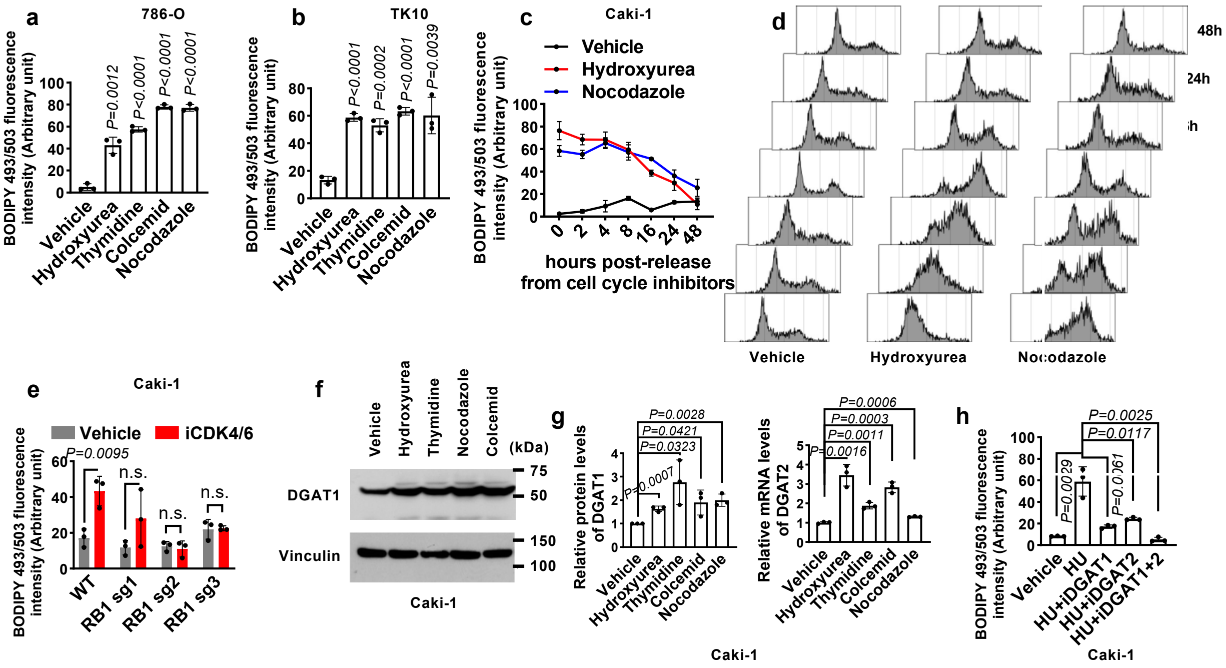

Supplementary Fig. 3. Cell-cycle arrest induces lipid droplet accumulation. a and b, The relative intensities of BODIPY 493/503 staining in 786-O (**a**) and TK10 (**b**) cells treated with cell-cycle inhibitors for 48 h. **c and d**. Caki-1 cells were treated with either vehicle, hydroxyurea or nocodazole for 24 h and then released for the indicated time points. BODIPY 493/503 staining was used to assess changes in lipid droplet accumulation over time after released from cell cycle inhibitors (**c**) and PI-staining was used to analyze the cell cycle (**d**). **e,** The relative intensities of BODIPY 493/503 staining in WT and RB1 sgRNA (sg1, sg2, and sg3)-infected Caki-1 cells treated with and without 2 μM iCDK4/6 for 48 h. **f,** Representative immunoblot of DGAT1 expression in Caki-1 cells treated with cell-cycle inhibitors for 48 h and the average relative DGAT1 protein levels normalized to vinculin protein levels from three independent experiments. **g,** The relative DGAT2 expression levels in Caki-1 cells treated with cell-cycle inhibitors for 48 h. **h,** The relative intensities of BODIPY 493/503 staining in Caki-1 cells given the indicated treatments for 48 h. HU, hydroxyurea (0.3 mM). Mean (± SD) values are shown. *n* = 3. *n* indicates

independent repeats (an unpaired, two-tailed *t*-test). n. s. not significant. Source data are provided as a Source Data file.

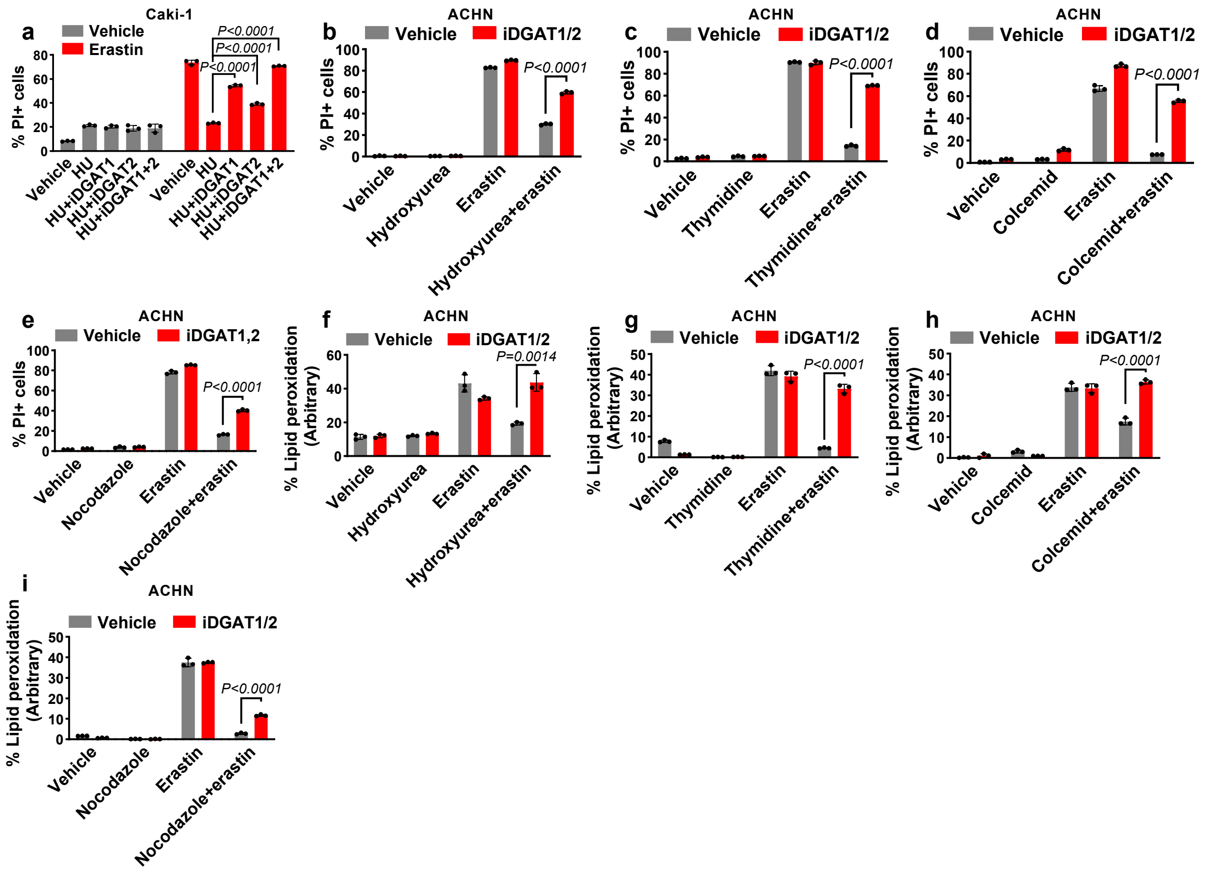

Supplementary Fig. 4. Triglycerides protects cells from ferroptosis. a, Populations of PI-positive Caki-1 cells pretreated with hydroxyurea together with the indicated DGAT inhibitors followed by 2 μM erastin for 18 h. **b-e**, Populations of PI-positive ACHN cells treated with 5 μM erastin for 24 h. Cells were pretreated with a vehicle or iDGAT1/2 for 24 h with 0.5 mM hydroxyurea (**b**), 2.5 mM thymidine (**c**), 0.035 μg/ml colcemid (**d**), or 200 nM nocodazole (**e**) for 24 h. **f-i**, Lipid peroxidation measurement in ACHN cells treated with 2 μM erastin for 18 h. Cells were pretreated with a vehicle or iDGAT1/2 together with 0.5 mM hydroxyurea (**g**), 2.5 mM thymidine (**g**), 0.035 μg/ml colcemid (**h**), or 200 nM nocodazole (**i**) for 24 h. Mean (± SD) values are shown. *n* = 3. *n* indicates independent repeats (two-tailed *t*-test). Source data are provided as a Source Data file.

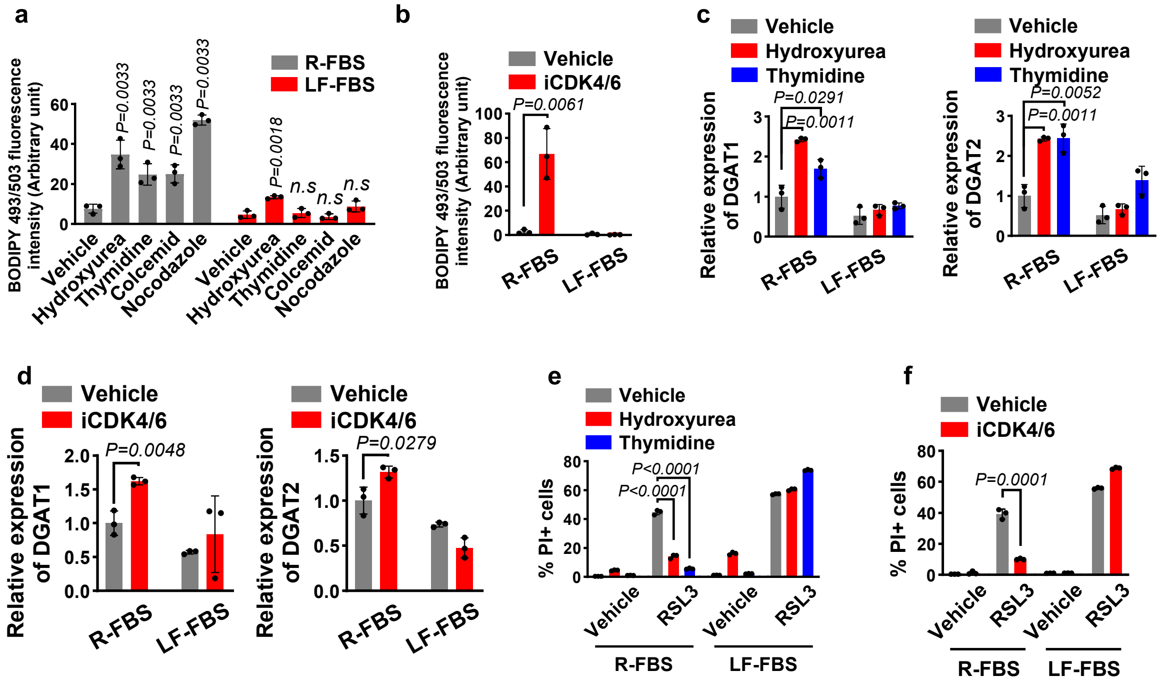

Supplementary Fig. 5. Ferroptosis resistance in cell cycle-arrested cells largely abolished with lipoprotein-free fetal bovine serum. a and b, The relative intensities of BODIPY 493/503 staining in Caki-1 cells. Cells were cultured with either regular-fetal bovine serum (R-FBS) or lipoprotein-free fetal bovine serum (LF-FBS) and treated with cell-cycle inhibitors for 30 h. **c and d**, The relative DGAT1 and DGAT2 expression levels in Caki-1 cells cultured with the indicated fetal bovine serum and treated with cell-cycle inhibitors for 30 h. **e and f,** Quantification of PI-positive dead Caki-1 cells using flow cytometry after 8 h of pretreatment with the indicated fetal bovine serum and cell cycle inhibitors for 8 h, followed by treatment with 50 nM RSL3 for 16h. Mean (± SD) values are shown. *n* = 3. *n* indicates independent repeats (unpaired, two-tailed *t*-test). n. s. not significant. Source data are provided as a Source Data file.

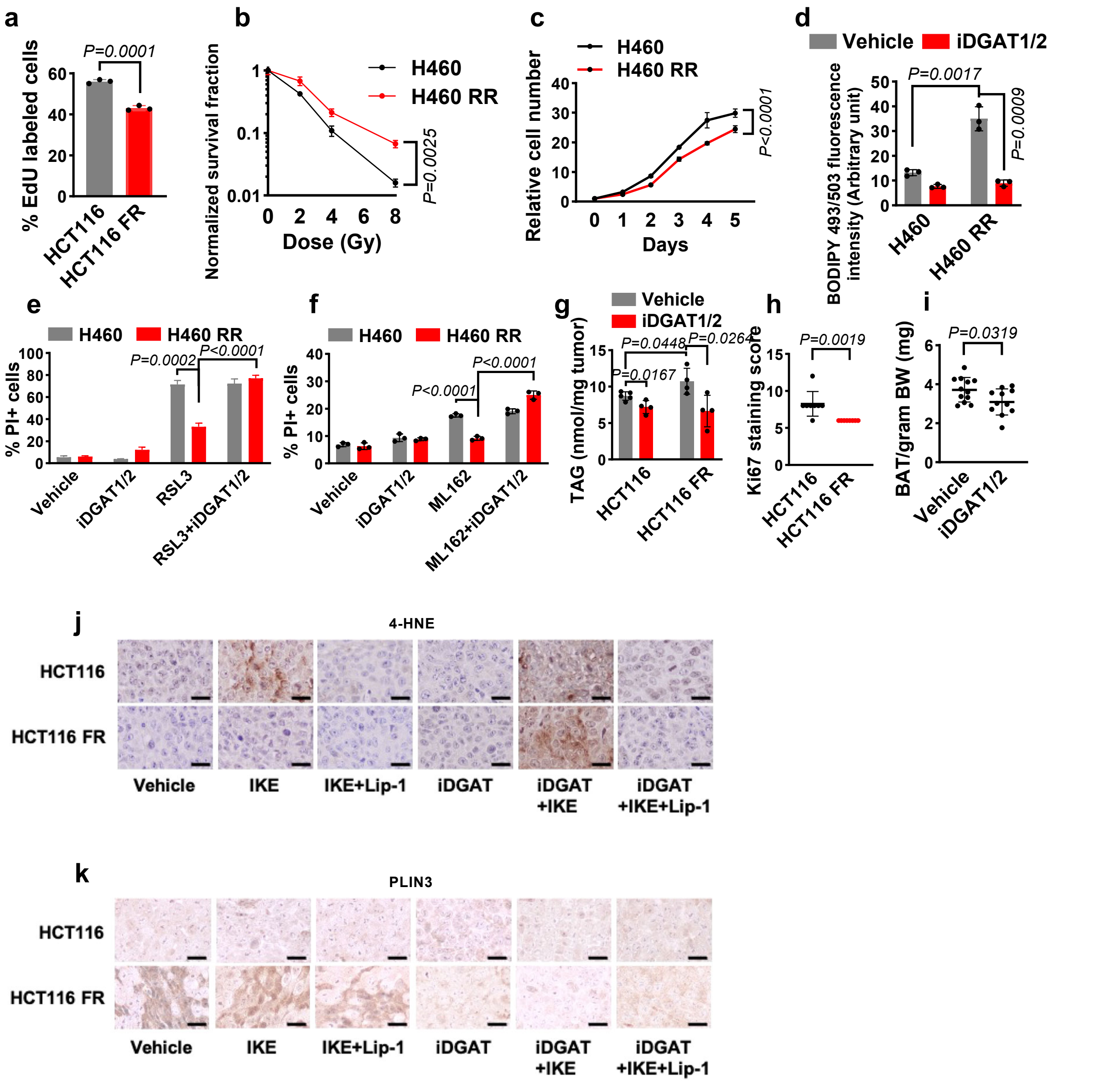

Supplementary Fig. 6. Treatment of slow-cycling therapy-resistant cells. a, Populations of EdU-positive labeled HCT116 and HCT116 FR cells measured using flow cytometry after a 1-h EdU pulse. **b,** Clonogenic survival curves for H460 and H460 RR cells exposed to radiation at different doses. Cell colonies were counted after 2 weeks of ionizing radiation exposure, and the numbers of survival fraction were normalized to those of unirradiated control cells. **c,** H460 and H460 RR cell growth measured for 5 days. **d,** The relative intensities of BODIPY 493/503 staining

in H460 and H460 RR cells treated with iDGAT1/2 for 48 h. **e and f,** The population of PI-positive cells treated with 10 μM RSL3 and iDGAT1/2 (**e**) and 5 μM ML162 and iDGAT1/2 (**f**) for 20 h. **g,** Triglyceride (TAG) levels measured in tumors from xenograft models with the indicated treatments. $n = 4$, except HCT116 vehicle ($n = 5$). **h,** Immunochemical scoring of Ki-67 staining in HCT116 and HCT116 FR xenograft tumors treated with a vehicle. $n = 8$ tumors. **i,** The weights of brown adipose tissues normalized to mouse body weight in vehicle- and iDGAT1/2-treated mice. $n = 12$ (vehicle), $n = 11$ (iDGAT1/2) mice. Mean (± SD) values are shown. **j and k**, Representative images of 4-HNE (**j**) and PLIN3 (**k**) staining of HCT116 and HCT116 FR xenograft tumors with the indicated treatments. Scale bars, 20 μm. Mean (± SD) values are shown. $n = 3$. *n* indicates independent repeats, except g, h, and i. unpaired, two-tailed *t*-test. *P* values were calculated using two-way ANOVA (**b and c**) or an unpaired, two-tailed *t*-test. Source data are provided as a Source Data file.

Supplementary Table

Supplementary Table 1. Cell-cycle inhibitor treatment conditions

Cell line	Cell number 12-well plate	Hydroxyurea (mM)	Thymidine (mM)	Colcemid (μg/ml)	Nocodazole (nM)	Erastin (μM, hours; for cell death)	Erastin (μM, hours; for lipid peroxidation)
Caki-1	9.0×10^4	0.3	1	0.035	200	2, 18	2, 8
ACHN	1.5×10^5	0.5	2.5	0.035	200	5, 24	2, 18
HT1080	1.0×10^5	0.5	2	0.020	100	10, 24	5, 18
A375	1.0×10^5	0.5	2	0.035	200	20, 24	10, 18
MEFs	8.0×10^4	0.1	2	0.035	200	2, 16	2, 8
786-O	1.0×10^5	0.5	2	0.035	200	10, 24	5, 18
TK10	1.0×10^5	0.5	2	0.035	200	10, 24	5, 18
