# Supplementary material for: Cell cycle arrest induces lipid droplet formation and confers ferroptosis resistance
Source: Nat Commun. 2024 Jan 2;15:79. doi: 10.1038/s41467-023-44412-7 (PMC10761718; doi:10.1038/s41467-023-44412-7)
Supplement: Supplementary file 4 — Reporting Summary [file 41467_2023_44412_MOESM4_ESM.pdf]

nature portfolio

Corresponding author(s): Boyi Gan
Last updated by author(s): Nov 27, 2023

Reporting Summary

Nature Portfolio wishes to improve the reproducibility of the work that we publish. This form provides structure for consistency and transparency in reporting. For further information on Nature Portfolio policies, see our Editorial Policies and the Editorial Policy Checklist.

Statistics

For all statistical analyses, confirm that the following items are present in the figure legend, table legend, main text, or Methods section.

n/a	Confirmed	
☐	☒	The exact sample size (*n*) for each experimental group/condition, given as a discrete number and unit of measurement
☐	☒	A statement on whether measurements were taken from distinct samples or whether the same sample was measured repeatedly
☐	☒	The statistical test(s) used AND whether they are one- or two-sided<br>*Only common tests should be described solely by name; describe more complex techniques in the Methods section.*
☒	☐	A description of all covariates tested
☒	☐	A description of any assumptions or corrections, such as tests of normality and adjustment for multiple comparisons
☐	☒	A full description of the statistical parameters including central tendency (e.g. means) or other basic estimates (e.g. regression coefficient) AND variation (e.g. standard deviation) or associated estimates of uncertainty (e.g. confidence intervals)
☐	☒	For null hypothesis testing, the test statistic (e.g. *F*, *t*, *r*) with confidence intervals, effect sizes, degrees of freedom and *P* value noted<br>*Give P values as exact values whenever suitable.*
☒	☐	For Bayesian analysis, information on the choice of priors and Markov chain Monte Carlo settings
☒	☐	For hierarchical and complex designs, identification of the appropriate level for tests and full reporting of outcomes
☒	☐	Estimates of effect sizes (e.g. Cohen's *d*, Pearson's *r*), indicating how they were calculated

Our web collection on statistics for biologists contains articles on many of the points above.

Software and code

Policy information about availability of computer code

Data collection	Flow cytometry data were acquired using an Attune NxT flow cytometer (Thermo Fisher Scientific). Fluorescent images were captured using a confocal microscope (LSM 880, Zeiss). Optical density intensities were measured using a Synergy 2 microplate reader (BioTek).
Data analysis	GraphPad Prism 9 was used for bar graphs output and statistical analysis; FlowJo V10 was used for flow cytometry data analysis. The lipidomic data were analyzed using the MAVEN software suite. The LC-MS/MS data were analyzed using Thermo Scientific Lipidsearch software (version 5.1) and in-house-written R scripts.

For manuscripts utilizing custom algorithms or software that are central to the research but not yet described in published literature, software must be made available to editors and reviewers. We strongly encourage code deposition in a community repository (e.g. GitHub). See the Nature Portfolio guidelines for submitting code & software for further information.

Data

Policy information about availability of data

All manuscripts must include a data availability statement. This statement should provide the following information, where applicable:

- Accession codes, unique identifiers, or web links for publicly available datasets
- A description of any restrictions on data availability
- For clinical datasets or third party data, please ensure that the statement adheres to our policy

The mass spectrometry-based lipidomics data generated in this study have been deposited in the Zenodo database under accession code 10059819 (https://

zenodo.org/records/10059819). All data supporting the findings of this study are available within the paper and Source Data file. Source Data are provided with this paper.

Human research participants

Policy information about studies involving human research participants and Sex and Gender in Research.

Reporting on sex and gender	N/A
Population characteristics	N/A
Recruitment	N/A
Ethics oversight	N/A

Note that full information on the approval of the study protocol must also be provided in the manuscript.

Field-specific reporting

Please select the one below that is the best fit for your research. If you are not sure, read the appropriate sections before making your selection.

☒ Life sciences ☐ Behavioural & social sciences ☐ Ecological, evolutionary & environmental sciences

For a reference copy of the document with all sections, see nature.com/documents/nr-reporting-summary-flat.pdf

Life sciences study design

All studies must disclose on these points even when the disclosure is negative.

Sample size	No sample size calculations were performed. The sample size of the experiments was chosen based on previous experience in the lab.
Data exclusions	No samples or animals were excluded from the data analyses.
Replication	Multiple independent repeats were included for related experiments. Each experiment was performed for at least twice to make sure similar results are reproducible.
Randomization	4-6-week-old female nude mice were chosen as xenograft hosts and randomly allocated into experimental groups. In cell-based experiments, randomization was unnecessary due to the pre-treatment of cells or inherent differences in genotypes.
Blinding	For cell-based experiments, western blotting, immunostaining and FACS, cell types were known when prepare the samples or start to treat cells at the beginning of experiments, and data collection was conducted blindly. Measurement for cell viability, FACS, photo capture and histological analysis were performed by different individuals who were blinded to the experimental groups. Mass spectrometry analysis was blinded prior to analysis.

Reporting for specific materials, systems and methods

We require information from authors about some types of materials, experimental systems and methods used in many studies. Here, indicate whether each material, system or method listed is relevant to your study. If you are not sure if a list item applies to your research, read the appropriate section before selecting a response.

Materials & experimental systems

n/a	Involved in the study	
☐	☒	Antibodies
☐	☒	Eukaryotic cell lines
☒	☐	Palaeontology and archaeology
☐	☒	Animals and other organisms
☒	☐	Clinical data
☒	☐	Dual use research of concern

Methods

n/a	Involved in the study	
☒	☐	ChIP-seq
☐	☒	Flow cytometry
☒	☐	MRI-based neuroimaging

Antibodies

Antibodies used	phospho-Rb ([S780] Cell Signaling Technology; 9307, 1:1,000), RB1 (Cell Signaling Technology; 9309, 1:1,000), CDK1 (Cell Signaling

nature portfolio | reporting summary
March 2021

Antibodies used	Technology; 9116, 1:1,000), ACSL4 (Santa Cruz Biotechnology; sc-271800, 1:1,000), GPX4 (R&D Systems; MAB5457, 1:1,000), SLC7A11 (Cell Signaling Technology; 12691, 1:1,000), FSP1 (Santa Cruz Biotechnology; sc-377120, 1:300), DHODH (Proteintech; 14877-1-AP, 1:1,000), DGAT1 (Santa Cruz Biotechnology; sc-271934, 1:300), vinculin (Sigma; V4505, 1:50,000), and tubulin (Sigma; T9026, 1:10,000), Goat anti-rabbit IgG secondary antibody (Thermo Scientific; 31460, 1:5,000), Goat anti-mouse IgG secondary antibody (Proteintech; SA00001-1, 1:5,000). 4-HNE (Abcam; #ab46545, 1:400), PLIN3 (Abcam; #ab47638, 1:300), and Ki-67 (Cell Signaling Technology; #9027, 1:400).
Validation	All antibodies used in our study have been validated and detailed information could be found on the website from manufactures as listed below.<br>phospho-Rb, https://www.cellsignal.com/products/primary-antibodies/phospho-rb-ser780-antibody/9307; Rb1, https://www.cellsignal.com/products/primary-antibodies/rb-4h1-mouse-mab/9309; CDK1, https://www.cellsignal.com/products/primary-antibodies/cdc2-poh1-mouse-mab/9116; ACSL4, https://www.scbt.com/p/acsl4-antibody-a-5?requestFrom=search; GPX4, https://www.rndsystems.com/products/human-mouse-rat-glutathione-peroxidase-4-gpx4-antibody-565320_mab5457; SLC7A11, https://www.cellsignal.com/products/primary-antibodies/xct-slc7a11-d2m7a-rabbit-mab/12691?site-search-type=Products&N=4294956287&Ntt=slc7a11&fromPage=plp; FSP1, https://www.scbt.com/p/amid-antibody-b-6?requestFrom=search; DHODH, https://www.ptglab.com/products/DHODH-Antibody-14877-1-AP.htm; DGAT1, https://www.scbt.com/p/dgat1-antibody-a-5?requestFrom=search; Vinculin, https://www.sigmaaldrich.com/US/en/product/sigma/v4505; Tubulin, https://www.sigmaaldrich.com/US/en/product/sigma/t9026; Goat anti-rabbit IgG secondary antibody, https://www.fishersci.com/shop/products/goat-anti-rabbit-igg-h-l-secondary-antibody-hrp-invitrogen/PI32460; Goat anti-mouse IgG secondary antibody, https://www.ptglab.com/products/HRP-conjugated-Affinipure-Goat-Anti-Mouse-IgG-H-L-secondary-antibody.htm; 4-HNE, https://www.abcam.com/products/primary-antibodies/4-hydroxynonenal-antibody-ab46545.html; PLIN3, https://www.abcam.com/products/primary-antibodies/perilipin-3tip47-antibody-ab47638.html; Ki-67, https://www.cellsignal.com/products/primary-antibodies/ki-67-d2h10-rabbit-mab-ihc-specific/9027.

Eukaryotic cell lines

Policy information about cell lines and Sex and Gender in Research

Cell line source(s)	The Caki-1, ACHN, HT1080, A375, 786-O, HCT116, H460, and T47D cell lines were obtained from the ATCC. The T47D PR cell line was obtained from Dr. Khandan Keyomarsi (MD Anderson Cancer Center). The TK10 cell line was obtained from Dr. Gordon Mills (MD Anderson Cancer Center). Primary MEFs were established from embryos at embryonic day 13.5 and immortalized via infection with SV40 large T antigen.
Authentication	Cell line were not authenticated.
Mycoplasma contamination	All cell lines tested negative for mycoplasma contamination.
Commonly misidentified lines (See ICLAC register)	No ICLAC cell line was used in this study.

Animals and other research organisms

Policy information about studies involving animals; ARRIVE guidelines recommended for reporting animal research, and Sex and Gender in Research

Laboratory animals	4-6-week-old female nude mice were purchased from the Experimental Radiation Oncology Breeding Core Facility at MD Anderson Cancer Center. Mice were housed under specific-pathogen-free conditions with a 12 h light–12 h dark cycle. The ambient temperature was 21–23°C, with 45% humidity and the mice had ad libitum access to water and food.
Wild animals	No wild animals involved in this study.
Reporting on sex	Female mice were used for ease of handling in this study. Sex was not a factor considered in the study design.
Field-collected samples	No sample collected from field was used in this study.
Ethics oversight	All the xenograft model experiments were conducted in compliance with a protocol approved by the institutional Animal Care and Use Committee and the Institutional Review Board at The University of Texas MD Anderson Cancer Center. The study adheres to all relevant ethical regulations pertaining to animal research.

Note that full information on the approval of the study protocol must also be provided in the manuscript.

Flow Cytometry

Plots

Confirm that:

☒ The axis labels state the marker and fluorochrome used (e.g. CD4-FITC).

☒ The axis scales are clearly visible. Include numbers along axes only for bottom left plot of group (a 'group' is an analysis of identical markers).

☒ All plots are contour plots with outliers or pseudocolor plots.

☒ A numerical value for number of cells or percentage (with statistics) is provided.

Methodology

Sample preparation	For cell death analysis, collected cells were stained with 5 µg/ml propidium iodide (PI, Roche, 11348639001) and the percentage of PI-positive dead cell population was analyzed by the flow cytometer, Attune NxT flow cytometer (Thermo Fisher Scientific) with a BL2 detector. For lipid peroxidation and lipid droplet, cells were incubated with 5 µM of C11-BODIPY 581/591 (Invitrogen, D3861) and 2 µM of BODIPY 493/503 (Invitrogen, D3922) at 37 °C, respectively. Then, cells were collected and subjected to flow cytometry analysis by he flow cytometer, Attune NxT flow cytometer (Thermo Fisher Scientific) with a BL1 detector.
Instrument	Attune NxT flow cytometer (Thermofisher)
Software	Attune NxT software was used for data collection and FlowJo_V10 software was used for data analysis.
Cell population abundance	At least 5,000 cells were analyzed for data collection.
Gating strategy	Initial cell population gating (SSC-Area VS SSC-Height) was adopted to make sure doublet exclusion and only single cell was used for analysis. An identical cell gating strategy was applied to all samples analyzed at the same time.

☐ Tick this box to confirm that a figure exemplifying the gating strategy is provided in the Supplementary Information.
